# Supplementary material for: Content-rich biological network constructed by mining PubMed abstracts
Source: BMC Bioinformatics. 2004 Oct 8;5:147. doi: 10.1186/1471-2105-5-147 (PMC528731; doi:10.1186/1471-2105-5-147)
Supplement: Additional File 2 — The original results of the above study (non-essential files are deleted to keep the file size under the limit set by BMC bioinformatics). [file 1471-2105-5-147-S2.bz2 › chilibotAdditionalFile2/dip05/631ID10026281E11018/html/ETFA_ETFB.html]

 


 **ETFA** and **ETFB** 
  
Found 7 abstracts in PubMed, retrieved 05.  
 

 What does Google say? 
 PDF only 
| .edu only 

---

**Interactive relationship** (e.g. stimulation, inhibition, etc)

**Inhibitory relationship**- Another neonatal onset patient showed normal synthesis of beta ETF  [ **ETFB** ]  but decreased synthesis of alpha ETF  [ **ETFA** ] .  Ref: 1430199 J Clin Invest, 1992
**Neutral relationship**

**Non-interactive relationship** (e.g. studied together, co-existance, homology, etc.)

- Open reading frames  **etfB**  and  **etfA** , located downstream of bcd, encode 27.2 and 36.1 kDa proteins, respectively, and show homology with the fixAB genes and the alpha and beta subunits of the electron transfer flavoprotein.  Ref: 8655474 J Bacteriol, 1996
- Three open reading frames bcd,  **etfB** , and  **etfA**  are located between crt and hbd.  Ref: 8655474 J Bacteriol, 1996
- Assignment of Etfdh,  **Etfb** , and  **Etfa**  to chromosomes, and 13 the mouse homologs of genes responsible for glutaric acidemia type II in human.  Ref: 8617498 Genomics, 1996
- We used cDNA probes for the Etfdh,  **Etfb** , and  **Etfa**  genes to determine localization of these mouse genes to chromosomes, and 13.  Ref: 8617498 Genomics, 1996
- We report the genomic structures of the  **ETFA** ,  **ETFB** , and ETFDH genes and the identification and characterization of seven novel and three previously reported disease causing mutations.  Ref: 12815589 Hum Mutat, 2003
